# Phosphate‐limited ocean regions select for bacterial populations enriched in the carbon–phosphorus lyase pathway for phosphonate degradation

**DOI:** 10.1111/1462-2920.14628

**Published:** 2019-05-27

**Authors:** Oscar A. Sosa, Daniel J. Repeta, Edward F. DeLong, Mohammad D. Ashkezari, David M. Karl

**Affiliations:** ^1^ Daniel K. Inouye Center for Microbial Oceanography: Research and Education University of Hawai‘i at Mānoa Honolulu HI 96822 USA; ^2^ Department of Marine Chemistry and Geochemistry Woods Hole Oceanographic Institution Woods Hole MA 02540 USA; ^3^ School of Oceanography University of Washington Seattle WA 98105 USA

## Abstract

In tropical and subtropical oceanic surface waters phosphate scarcity can limit microbial productivity. However, these environments also have bioavailable forms of phosphorus incorporated into dissolved organic matter (DOM) that microbes with the necessary transport and hydrolysis metabolic pathways can access to supplement their phosphorus requirements. In this study we evaluated how the environment shapes the abundance and taxonomic distribution of the bacterial carbon–phosphorus (C–P) lyase pathway, an enzyme complex evolved to extract phosphate from phosphonates. Phosphonates are organophosphorus compounds characterized by a highly stable C–P bond and are enriched in marine DOM. Similar to other known bacterial adaptions to low phosphate environments, C–P lyase was found to become more prevalent as phosphate concentrations decreased. C–P lyase was particularly enriched in the Mediterranean Sea and North Atlantic Ocean, two regions that feature sustained periods of phosphate depletion. In these regions, C–P lyase was prevalent in several lineages of *Alphaproteobacteria* (*Pelagibacter*, SAR116, *Roseobacter* and *Rhodospirillales*), *Gammaproteobacteria,* and *Actinobacteria*. The global scope of this analysis supports previous studies that infer phosphonate catabolism via C–P lyase is an important adaptive strategy implemented by bacteria to alleviate phosphate limitation and expands the known geographic extent and taxonomic affiliation of this metabolic pathway in the ocean.

## Introduction

Phosphorus (P) is essential to all living organisms for growth, energetics and nucleic acid synthesis. P is most readily available to microorganisms as inorganic phosphate (Pi). However, vast ocean regions in tropical and subtropical latitudes are characterized by persistent low Pi concentrations, a condition that has been shown to limit microbial productivity (Krom *et al*., 1991; Cotner *et al*., 1997; Wu *et al*., 2000; Thingstad *et al*., 2005; Moore *et al*., 2013). The increasingly recognized importance of Pi availability to the ecology of marine plankton (Karl *et al*., 2001; Ammerman *et al*., 2003; Dyhrman *et al*., 2007) and the detailed understanding of the physiological response of *Escherichia coli* and other reference organisms to Pi deficiency have set the stage to investigate the genetic adaptations and physiological acclimations marine microorganisms display to cope with P scarcity.

A characteristic feature of the bacterial Pi deficiency response is the expression of enzymes and pathways that have evolved to extract P from organic compounds (Wackett *et al*., 1987; Wanner, 1998). In oligotrophic oceanic environments, the inventory of dissolved organic phosphorus (DOP) compounds typically exceeds the pool of free Pi and the P stored in particulate organic matter (Björkman and Karl, 2003; Lomas *et al*., 2010). Phosphate esters are the largest identifiable chemical class of P‐containing compounds in marine dissolved organic matter (DOM), followed by phosphonates, reduced P compounds (P oxidation state of +3) with a direct C–P electron bond (Kolowith *et al*., 2001; Young and Ingalls, 2010). Numerically dominant bacterial groups in marine surface waters express enzymes known as alkaline phosphatases (APases) to hydrolyze phosphate esters under Pi stress (Kathuria and Martiny, 2010). Similarly, microorganisms have evolved specialized enzymatic pathways designed to break the C–P bond of phosphonates in order to assimilate P (Quinn *et al*., 2007; White and Metcalf, 2007). These pathways are widespread in marine microorganisms, especially among the *Proteobacteria* (Villarreal‐Chiu *et al*., 2012) and their distribution seems to vary across ecosystems (Martinez *et al*., 2010).

Among the known phosphonate degradation pathways, bacterial C–P lyase plays a unique role in the biogeochemistry of the ocean. C–P lyase is a multi‐enzyme complex encoded by a set of co‐transcribed genes (Metcalf and Wanner, 1993; Seweryn *et al*., 2015) whose expression is controlled by Pi concentration as part of the Pho regulon that responds to Pi starvation in bacteria (Chen *et al*., 1990). C–P lyase can degrade a broad range of phosphonate substrates (Kononova and Nesmeyanova, 2002; White and Metcalf, 2007) including alkyl phosphonates, the major class of phosphonates identified in marine high molecular weight (HMW) DOM (Repeta *et al*., 2016). The C–P bond cleavage reaction of alkyl phosphonates by the C–P lyase pathway is encoded by the gene *phnJ* and is essential for organisms to recover Pi from phosphonates (Kamat *et al*., 2013). This reaction also releases the attached alkyl group of the phosphonate as the corresponding alkane (Kamat *et al*., 2013; Seweryn *et al*., 2015). This latter property has linked C–P lyase degradation of methylphosphonate, one of the most abundant alkylphosphonates found in HMW DOM, to the slight supersaturation of methane observed throughout marine surface waters (Karl *et al*., 2008; Repeta *et al*., 2016), a phenomenon known as the marine methane paradox (Kiene, 1991).

In this study, we used a metagenomic approach to compare Pi concentration and other environmental parameters to the abundance of genes encoding enzymes that form the C–P lyase pathway for phosphonate degradation and compared this to other pathways implicated in phosphonate catabolism, DOP turnover and P acquisition. These included the phosphonate hydrolysis pathways encoded by *phnX* and *phnA* which mediate the degradation of phosphonacetaldehyde and phosphonacetate, respectively (Quinn *et al*., 2007); the APases encoded by *phoX*, *phoD* and *phoA*; and the high‐affinity Pi membrane transport system proteins encoded by *pstABCS*. Two additional genes, *phoB* and *plcP*, were included in the analysis to serve as markers of Pi deficiency. *phoB* encodes a transcription activator of the Pi starvation response in bacteria (Wanner and Chang, 1987) and *plcP* encodes a phospholipase enzyme involved in cell membrane phospholipid substitution to save P and is prevalent in Pi limited ocean regions (Carini *et al*., 2015; Sebastián *et al*., 2016). We also used phylogenetic information to identify which bacterial groups encode C–P lyase across different oceanic regions. Through this approach, we evaluate how Pi concentration shapes the distribution and abundance of C–P lyase in marine bacterial communities relative to other P acquisition pathways and how this interaction may in turn influence the cycling of DOM phosphonates in marine surface waters.

## Results

**Figure 1:**
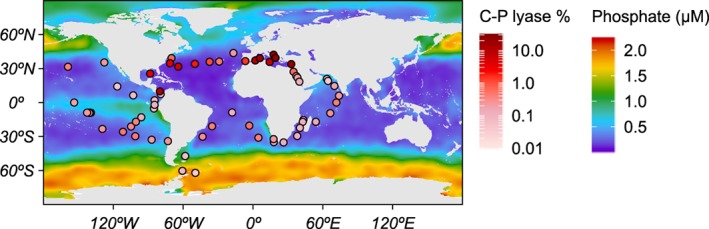
Distribution of C–P lyase and phosphate in marine surface waters. The circles represent metagenomic sampling locations of *Tara* Oceans in surface waters (5 m depth) and the colour scale indicates the percentage of organisms possessing the C–P lyase pathway gene *phnJ*. The map background colour gradient represents the mean annual inorganic phosphate concentration in surface waters obtained from the WOA 2009 mean annual climatology.

An exploratory heat map of C–P lyase gene *phnJ* relative abundance in samples collected at 5 m depth indicated that C–P lyase was enriched in Pi‐depleted ocean regions (Fig. 1). Of the environmental parameters tested, Pi concentration had the strongest negative correlation with the relative abundance of C–P lyase in the epipelagic zone (EPZ), followed by silicate and inorganic nitrogen (N, nitrate plus nitrite) (Table 1). Using the mean annual Pi concentration data from the World Ocean Atlas (WOA) and the monthly Pi climatology of Pelagic Interactions Scheme for Carbon and Ecosystem Studies (PISCES) improved this correlation (Table 1). In addition, dissolved iron (Fe) and the dissolved Fe:Pi molar ratio obtained from PISCES had a significant positive correlation with C–P lyase relative abundance in the EPZ (Table 1).

**Table 1:** Correlation analyses of *phnJ* relative abundance and environmental parameters from *Tara* Oceans, the World Ocean Atlas (WOA) mean annual climatology, and the Pelagic Interactions Scheme for Carbon and Ecosystem Studies (PISCES) monthly climatology.

	Epipelagic		Mesopelagic	
Parameter	*r*	*p*	*r*	*p*
Depth	−0.03	0.73	0.59	***
Oxygen	0.04	0.68	0.65	***
Temperature	−0.02	0.87	−0.41	*
Silicate	−0.26	**	−0.22	0.23
Phosphate	−0.31	***	−0.40	*
Nitrate plus nitrite	−0.23	*	−0.33	0.08
N:P	−0.10	0.31	0.06	0.74
WOA Phosphate	−0.44	***	–	–
WOA Nitrate	−0.29	**	–	–
WOA N:P	0.05	0.65	–	–
PISCES Oxygen	−0.01	0.94	–	–
PISCES Silicate	−0.38	***	–	–
PISCES Nitrate	−0.39	***	–	–
PISCES Phosphate	−0.54	***	–	–
PISCES Iron	0.58	***	–	–
PISCES Iron:Phosphate	0.44	***	–	–
PISCES Primary production	−0.09	0.34	–	–
PISCES Phytoplankton	−0.1	0.29	–	–
PISCES Chlorophyll	−0.06	0.53	–	–

Significance level: <0.001 (***), <0.01 (**), <0.05 (*).

**Figure 2:**
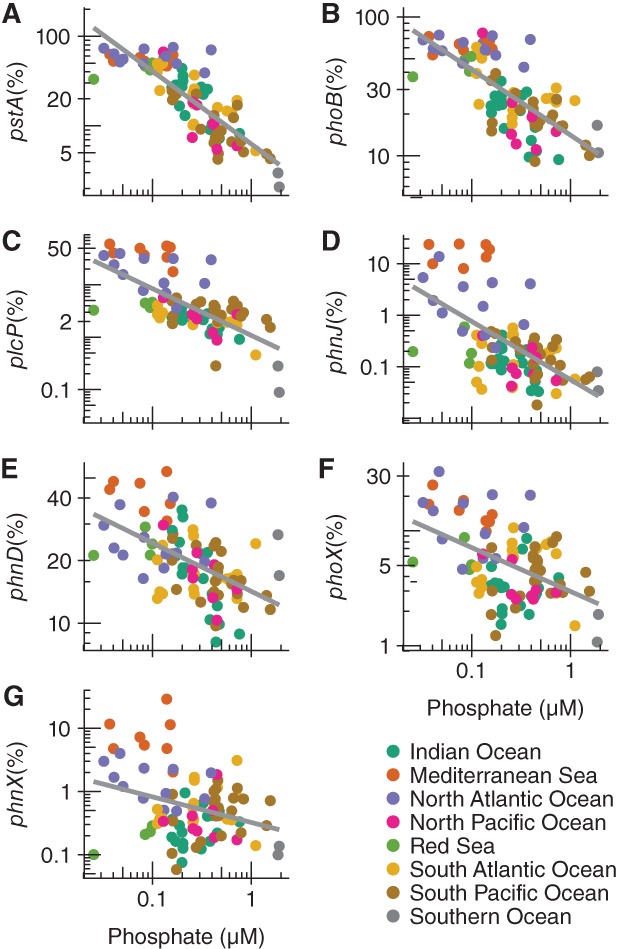
Phosphorus acquisition gene relative abundance with respect to phosphate concentration in the epipelagic zone. Gene abundance was normalized to *recA* and expressed as a percentage of organisms. Phosphate concentrations were obtained from the WOA. The relative abundance and phosphate axes were log_10_‐transformed. A. Linear model of high‐affinity Pi transport gene *pstA*. B. Pho regulon gene *phoB*. C. Phospholipase for membrane lipid remodelling gene *plcP*. D. C–P lyase gene *phnJ*. E. Phosphonate transport gene *phnD*. F. Alkaline phosphatase gene *phoX*. G. Phosphonoacetaldehyde hydrolase gene *phnX*. Data points are colour coded by ocean region. All linear regression models had a significant inverse relationship between gene relative abundance and phosphate concentration (*P* < 0.05).

In our regression model of log‐transformed *phnJ* relative abundance with two predictors, ocean region and log‐transformed Pi concentration from WOA, both were significant at the 0.001 level. To further evaluate the relationship between C–P lyase gene abundance and Pi concentration, we compared the Pi WOA mean annual climatology to the relative abundance of representative genes encoding alternate P acquisition pathways and Pi stress marker genes (Fig. 2). Of the genes tested, the abundance of high‐affinity Pi uptake system gene *pstA* had one of the highest negative correlation with Pi (*r* ^2^ = 0.67, *P* < 0.001), and occurred in approximately half of the organisms in the EPZ in the Mediterranean Sea (MS) and North Atlantic Ocean (NAO) (Fig. 2A). The slope of the relationship of *pstA* relative abundance with Pi was not significantly different from the high‐affinity Pi uptake genes *pstBCS* (*P* > 0.05; Supporting Information Table S1). The Pi stress marker genes *phoB* had the next strongest negative correlation with Pi (*r* ^2^ = 0.52, *P* < 0.001; Fig. 2B) after *pstABCS*, followed by the *plcP* gene for phospholipid substitution (*r* ^2^ = 0.45, *P* < 0.001; Fig. 2C). The C–P lyase catalytic gene *phnJ* had the fourth strongest negative relationship with Pi (*r* ^2^ = 0.35, *P* < 0.001; Fig. 2D). The slope of the regression of *phnJ* against Pi was not significantly different from the slope of the regressions of the C–P lyase catalytic genes *phnGHIKL* (*P* > 0.05; Supporting Information Table S1). The regression of the abundance of C–P lyase pathway catalytic genes against WOA Pi had the highest inverse slopes, followed by the regressions of Pi transport genes and *plcP* (Supporting Information Table S1). The phosphonate transport gene *phnD* (*r* ^2^ = 0.28, *P* < 0.01; Fig. 2E) and the APase encoded by *phoX* (*r* ^2^ = 0.23, *P* < 0.001; Fig. 2F) followed similar inverse relationships with Pi though not as robust as for C–P lyase pathway catalytic genes and high‐affinity Pi transport genes. The phosphonoacetaldehyde hydrolase encoded by *phnX* (*r* ^2^ = 0.08, *P* < 0.05) also had a significant, albeit weak, negative relationship with Pi (Fig. 2E). The remaining genes tested did not follow a significant negative relationship with Pi (*P* > 0.05; Supporting Information Table S1).

**Figure 3:**
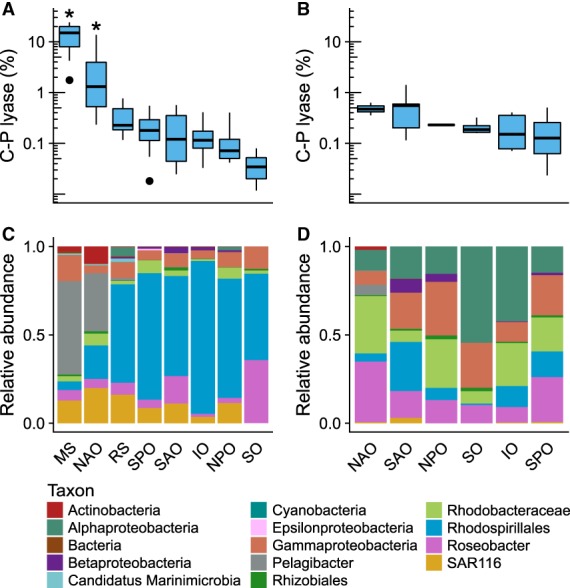
Abundance and taxonomic distribution of C–P lyase in representative ocean regions. A. C–P lyase in the epipelagic zone. B. C–P lyase in the mesopelagic zone. In each subfigure, the upper panel boxplots depict the distribution of the percentage of bacteria possessing C–P lyase gene *phnJ* in representative ocean regions. The ends of the box indicate the first and third quartiles. Whiskers extend up to 1.5 times the interquartile range. Solid black symbols denote outliers. Ocean regions are ranked in descending order by the average relative abundance of *phnJ*. Ocean regions indicated with (*) were significantly enriched with C–P lyase relative to all other regions (*P* < 0.05). The lower panel bar charts indicate the average proportion of *phnJ* sequence abundance accounted for by different taxa. Taxa are indicated in different colour bars. Bars indicated as higher level taxa denote the abundance of additional subgroups within that lineage.

Based on our analysis, the distribution of C–P lyase gene abundance was statistically distinct between oceanic regions in the EPZ [*X* ^*2*^(7, *N* = 118) = 66.7, *P* < 6.8 × 10^−12^] and was significantly enriched in the MS and the NAO (*P* < 0.05) relative to all other ocean regions (Fig. 3A). C–P lyase abundance was lower than the high‐affinity Pi transport system genes *pstABCS*, the Pi stress response gene *phoB* and the phospholipid substitution gene *plcP* and occurred in 2%–24% of organisms in the EPZ in the MS and 0.2%–14% in the NAO. Outside these regions, C–P lyase representation declined to 0.04%–0.4% of organisms (Fig. 3). The abundance of C–P lyase was comparable to that of APases in the MS and NAO. *phoX* was present on average in 16% and 13% of organisms and *phoD* in 22% and 16% of organisms respectively. Outside these regions, *phoX* and *phoD* abundance did not decline as drastically as C–P lyase, remaining in 9%–17% and 2%–9% of organisms respectively. C–P lyase abundance was also similar to the abundance of *phnX* which occurred on average in 8% of organisms in the MS and in 0.2%–2% of organisms in the EPZ outside this region.

In the mesopelagic zone (MPZ), C–P lyase relative abundance was significantly different across ocean basins [*X* ^*2*^(5, *N* = 32) = 11.5, *P* = 0.043]. However, unlike the disproportional abundance of C–P lyase in the EPZ in the MS and NAO, C–P lyase was not significantly enriched in the MPZ of any ocean region (*P* > 0.05) and occurred on average in 0.2%–0.7% of organisms (Fig. 3B). Depth and dissolved oxygen had strong positive correlations with C–P lyase relative abundance in the MPZ, while temperature and Pi had significant negative correlations (Table 1). While C–P lyase abundance declined in the MPZ relative to the EPZ, APases and other phosphonate degradation pathways remained well represented in the MPZ. *phoX* occurred on average in 12%–19% of organisms while *phoD* was present on average in 11%–25%. *phnX* was also well represented in the MPZ, occurring in 8%–20% of organisms. In turn, *phnA*, which encodes a phosphonoacetate hydrolase, occurred in 30%–57% of organisms in this region.

Based on our phylogenetic analysis, C–P lyase was present in multiple lineages of *Proteobacteria*, as well as in *Firmicutes* and *Actinobacteria*, and was particularly well represented in the *Alphaproteobacteria* (Supporting Information Fig. S1). The distinct increase of C–P lyase abundance in the MS and NAO was marked by an enrichment of sequences closely related to the C–P lyase of *Pelagibacterales* sp. HITCC7211, a representative of the SAR11 clade of *Alphaproteobacteria* (Carini *et al*., 2014). *Pelagibacter* C–P lyase contributed on average 47% and 30% of C–P lyase abundance in the MS and NAO sites, respectively, but was absent outside these regions (Fig. 3C). Another group of C–P lyase sequences that clustered with *Actinobacteria* sequences related to *Streptomyces* and *Mycobacteria* (Supporting Information Fig. S1; see also the expanded tree in the Supporting Information) was also prevalent in the EPZ in the MS and NAO but its abundance declined beyond these sites (Fig. 3).

C–P lyase sequences closely related to those found in the SAR116 clade, in the marine *Rhodobacteraceae* including the *Roseobacter* clade, and in several *Rhodospirillales* lineages of *Alphaproteobacteria* (Supporting Information Fig. S1) were also well represented in the EPZ in the MS and NAO and were distributed more broadly in the marine environment (Fig. 3C and D). In the North Pacific Subtropical Gyre (NPSG) we identified 38 unique *phnJ* genes from the Station ALOHA gene catalogue which were most similar to those of *Roseobacter* and *Rhodospirillales* (Supporting Information Fig. S1; see also the expanded tree in the Supporting Information). C–P lyase was also associated with *Deltaproteobacteria* of the order *Desulfovibrionales*, several groups of *Gammaproteobacteria* including the *Oceanospirillales*, *Vibrionales*, *Alteromonadales* and *Pseudomonadales*, as well as with the candidate phylum Marinimicrobia (Supporting Information Fig. S1). We also found C–P lyase genes similar to those of marine *Firmicutes*, *Cyanobacteria* and *Chloroflexi* but their abundance was relatively minor (Fig. 3C and D). In the MPZ, C–P lyase associated with *Gammaproteobacteria*, *Roseobacter* and other groups of *Alphaproteobacteria* which made up a larger fraction of C–P lyase abundance compared to the EPZ, while *Pelagibacter* and SAR116 sequences diminished at these depths (Fig. 3D).

**Figure 4:**
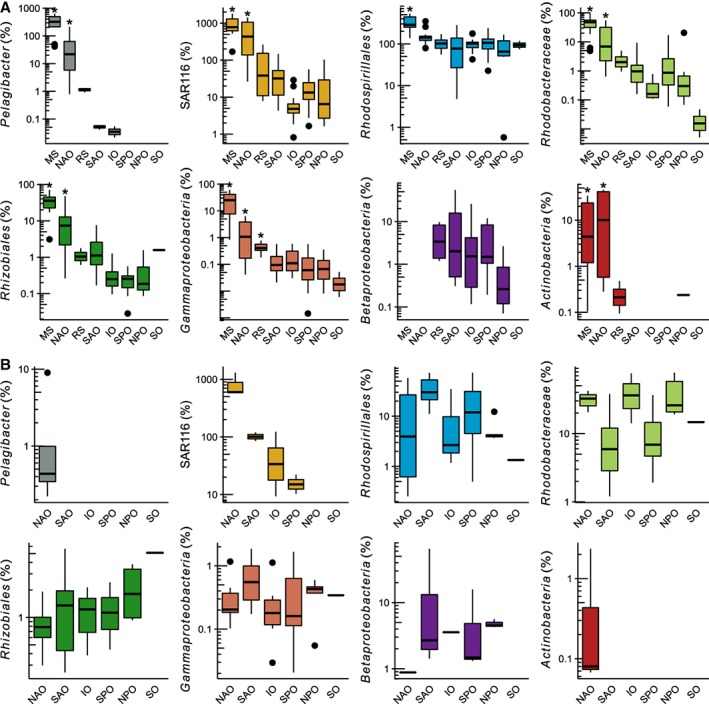
Taxon‐specific enrichment of C–P lyase. The data were derived from *Tara* Oceans samples of the 0.22 μm size fraction collected in (A) the epipelagic zone or in (B) the mesopelagic zone in different ocean regions: Mediterranean Sea (MS), North Atlantic Ocean (NAO), Red Sea (RS), South Atlantic Ocean (SAO), Indian Ocean (IO), South Pacific Ocean (SPO), North Pacific Ocean (NPO) and Southern Ocean (SO). The boxplots depict the distribution of the percentage of bacteria possessing the C–P lyase gene *phnJ*. The ends of the box indicate the first and third quartiles. Whiskers extend up to 1.5 times the interquartile range. Solid black symbols denote outliers. The boxplot colour scheme is based on the taxon bins in Fig. 3. The *Rhodobacteraceae* boxplot data include the *Rhodobacteraceae* and *Roseobacter* taxon bins in Fig. 3. Ocean regions indicated with (*) were significantly enriched with C–P lyase relative to all other regions (*P* < 0.05).

In the EPZ, the relative enrichment of C–P lyase in specific taxonomic groups varied significantly (*P* < 0.05) across ocean regions (Fig. 4A). For example, C–P lyase was significantly enriched in *Pelagibacter* in the MS and NAO relative to all other ocean regions (*P* < 0.05). On average, C–P lyase occurred in 44% of *Pelagibacter* in the NAO and in 340% in the MS (Fig. 4A). Values > 100% are potentially indicative of the presence of multiple gene copies per genome. A similar C–P lyase enrichment in the MS and NAO was found in the SAR116 clade, *Rhodobacteraceae*, *Rhizobiales,* and *Actinobacteria* (Fig. 4A). C–P lyase was prevalent in the *Rhodospirillales* across all ocean regions, occurring on average in 60%–330% of organisms and was significantly higher in the MS relative to other ocean regions. C–P lyase was also significantly enriched in the *Gammaproteobacteria* in the EPZ in the MS relative to other ocean regions. In turn, in the MPZ, the average relative enrichment of C–P lyase only varied significantly (*P* < 0.05) in *Pelagibacter*, the SAR116 clade and *Actinobacteria* (Fig. 4B). In the NAO, for example, C–P lyase occurred on average in ~2% of *Pelagibacter* organisms and in 0.4% of *Actinobacteria*. In some sampling locations in the NAO, C–P lyase representation exceeded one copy per cell in the SAR116 clade. However, in neither of these taxa was C–P lyase significantly enriched in the MPZ in a particular ocean region. Nevertheless, on average C–P lyase persisted in a considerable proportion of bacteria in the *Rhodobacteraceae* (11%–40% of organisms) and *Rhodospirillales* (1%–37%) across all ocean regions in the MPZ (Fig. 4B).

The mapping of *Tara* Oceans metagenomic reads to the genome of *Pelagibacterales* sp. strain HTCC721 and to *Tara* Oceans metagenome‐assembled genomes (MAGs) revealed consistent enrichment patterns of C–P lyase in these taxonomic groups (S2). Based on read mapping, C–P lyase occurred on average in 628% (~6 copies cell^−1^) of the *Pelagibacter* population represented by the HTCC7211 genome in MS surface waters (5 m depth samples), and in 45% in the NAO and declined below 0.1% outside these regions (S2). A similar enrichment was observed for *Pelagibacter* with C–P lyase in the deep chlorophyll maximum (DCM), 352% (~4 copies cell^−1^) in the MS and 14% in the NAO. Among the *Alphaproteobacteria* MAGs, C–P lyase was prevalent in the SAR116 clade, *Rhodospirillales* (*Thalassospira*, *Tistrella* and unclassified sublineages), *Rhodobacteraceae* (*Roseovarius*, *Roseobacter* and unclassified sublineages) and *Rhizobiales*. In some of these populations, C–P lyase was persistent across all ocean regions and depths (S2). Among the *Gammaproteobacteria* MAGs, C–P lyase was prevalent in members of the *Oceanospirillaceae* (*Salinicola* and *Halomonas*), some *Pseudomonas* and only in a small percentage of *Alteromonadales* (S2). A subset of *phn* operons representative of the C–P lyase pathway found in SAR116 clade, *Rhodospirillales*, *Oceanospirillales*, and Marinimicrobia MAGs are presented in the Supporting Information S3.

## Discussion

Microbes cope with nutrient limitation by modifying their cellular elemental requirements or by expanding the range of substrates they can metabolize to acquire the needed resource (Merchant and Helmann, 2012). In the ocean, Pi availability constitutes a major selective pressure that has shaped the genome content and adaptations exhibited by abundant bacterial populations (Coleman and Chisholm, 2010). In Pi‐limited ocean regions, for example sparing cellular P by replacing membrane phospholipids with non‐P lipids appears to be a common strategy used by phytoplankton, including abundant cyanobacteria like *Prochlorococcus* (Van Mooy *et al*., 2006, 2009), and heterotrophic bacteria (Carini *et al*., 2015; Sebastián *et al*., 2016) to economize the P available in the cell. The strong inverse relationship we observed between global ocean Pi concentration and the abundance of *phoB* of the Pi stress response regulatory system and *plcP* for cell membrane phospholipid substitution is consistent with these observations. The strong inverse correlations between Pi and pathways for Pi transport systems and pathways for transport and hydrolysis of bioavailable organic P compounds in DOM (phosphonates and phosphate esters) are also in agreement with their critical role in bacteria to cope with Pi limitation in the ocean (Dyhrman *et al*. 2007).

Our analyses revealed an enrichment of C–P lyase in bacterial communities in the EPZ in the MS and NAO, confirming the results of Coleman and Chisholm (2010) who found C–P lyase to be more prevalent in *Pelagibacter* populations in the North Atlantic Subtropical Gyre near Bermuda relative to the NPSG near Hawai‘i. The prevalence of genes encoding phosphonate transport proteins associated with *Pelagibacter* in the eastern MS has also been reported (Feingersch *et al*., 2010). Both studies attributed the enhanced abundance of C–P lyase pathway genes to low Pi concentration, a conclusion supported by our comparison of global C–P lyase abundance and Pi concentration data from *Tara* Oceans, WOA and PISCES. However, our comparison of the abundance and response of different Pi acquisition pathways and Pi stress markers to Pi concentration indicates that at the genome level, high‐affinity Pi uptake systems and phospholipid substitution are more prevalent adaptations than the C–P lyase pathway to cope with Pi scarcity in the EPZ (Fig. 2).

In our analysis, both Pi concentration and ocean region were good proxies for the enrichment of C–P lyase in the bacterial community consistent with the extended periods of Pi scarcity observed in NAO and MS surface waters (Krom *et al*., 1991; Wu *et al*., 2000; Cavendar‐Bares *et al*., 2001) which can ultimately limit the growth of heterotrophic bacteria (Thingstad and Rassoulzadegan, 1995; Cotner *et al*., 1997). C–P lyase abundance was also negatively correlated with inorganic N and silicate. However, given that N scarcity and silicate are not expected to play a regulatory or biochemical role in the C–P lyase pathway, these trends may reflect the enrichment of C–P lyase with Pi scarcity which coincides with other oligotrophic conditions. In the NAO, Pi limitation is in great part driven by the additional N available through N_2_ fixation stimulated by the aeolian Fe supply from the Sahara Desert (Wu *et al*. 2000), and through atmospheric deposition (rainfall and aerosols) of bioavailable N (Singh *et al*. 2013). In turn, in the MS, particularly in the eastern basin, Pi limitation arises due to the high N:P ratio of external inputs including to a large extent atmospheric N deposition and the inflow waters from the western MS (Krom *et al*. 2010). The MS also receives vast quantities of Fe from Sahara Desert dust which is reflected in the increase of dissolved Fe concentrations in surface waters (Guieu *et al*., 2002). However, in this region, N_2_ fixation is not considered to play a primary role in Pi limitation (Krom *et al*., 2010). The importance of Fe in Pi limitation may explain the positive correlation between Fe concentration and *phnJ* abundance (Table 1). However, a direct relationship may exist between the two. The C–P lyase pathway protein PhnJ catalytic mechanism requires Fe for a redox‐active Fe–sulfur (4Fe–4S) cluster for C–P bond cleavage (Kamat *et al*., 2013; Seweryn *et al*., 2015). In addition, in a field study in the NPSG, del Valle and Karl (2014) found that adding dissolved Fe to surface water microbial communities enhanced the conversion of methylphosphonate to methane. These results suggest that Pi scarcity, and perhaps Fe availability, are key factors controlling the abundance and distribution of C–P lyase in marine surface waters.

Despite experiencing very low Pi concentrations (< 20 nM) throughout the year, the DOP inventory in North Atlantic Subtropical Gyre surface waters is 4‐ to 7‐fold greater than Pi and particulate P (Torres‐Valdés *et al*., 2009; Lomas *et al*., 2010). Relative increases in APase activity and particulate organic P concomitant with DOP decline during the winter/spring phytoplankton bloom in this region were seen as indicative of enhanced DOP cycling (Lomas *et al*., 2010). Similarly, in the MS, particularly in the eastern basin where Pi concentrations < 10 nM can extend as deep as 450 m after winter mixing (Krom *et al*., 1991), enhanced APase activity (Zaccone *et al*., 2012) and net removal of DOP (Powley *et al*., 2017) reflect the critical role of DOP degradation in sustaining the bacterial P demand. PhoX and PhoD are prevalent APases in these regions (Sebastian and Ammerman, 2009; Kathuria and Martiny, 2010) and are likely involved in the high rates of DOP turnover. The comparable representation of C–P lyase and APases in the EPZ in MS and NAO indicates that phosphonates are a particularly important source of Pi to bacteria in these regions and that their degradation may contribute to the observed remineralization of DOP.

In the MPZ, on the other hand, C–P lyase abundance declined considerably relative to the EPZ and the negative relationship with Pi was not as robust as with other environmental parameters (Table 1). This result may be partly explained by the lack of *Tara* Oceans metagenomes sampled in the MS MPZ which is expected to be depleted in Pi relative to other nutrients (Krom *et al*., 1991) and enriched in C–P lyase. In contrast to the decline of C–P lyase in the MPZ, the phosphonate hydrolases encoded by *phnA* and *phnX* became relatively more abundant. This is consistent with previous surveys of phosphonate degradation functions and in support of the hypothesis that phosphonate degradation at these depths functions independently of Pi and is instead important to obtain C and N (Quinn *et al*., 2007; Martínez *et al*., 2010; Luo *et al*., 2011; Chin *et al*., 2018). Thus, the distribution of C–P lyase in the EPZ is strongly linked to Pi scarcity.

### *Diversity and distribution of bacteria encoding C–P lyase*

*Pelagibacter* populations possessing C–P lyase were prevalent in the EPZ in the MS and NAO. The occurrence of C–P lyase in this clade in the NAO (~45% of *Pelagibacter*) was consistent with a previous survey in the Sargasso Sea which found that up to ~52% of *Pelagibacter* carry the *phn* operon (Coleman and Chisholm, 2010). In the MS, however, we found that virtually all *Pelagibacter* carry C–P lyase as the occurrence of C–P lyase exceeded one copy per organism (> 100% relative abundance). Although C–P lyase was enriched in the numerically dominant *Pelagibacter* clade, we found that the increase of C–P lyase abundance in the MS and NAO was also driven by several lineages of *Alphaproteobacteria* and *Gammaproteobacteria*. Among the *Alphaproteobacteria* lineages, the enrichment of C–P lyase in the SAR116 clade and the *Rhodospirillales* had not been detected before. Similar to *Pelagibacter*, the SAR116 clade is an abundant member of the bacterial community and contains genes that encode functions characteristic of an oligotrophic lifestyle, such as the metabolism of one‐carbon organic compounds and the light‐driven proton pump proteorhodopsin (Oh *et al*., 2010). The *Rhodospirillales* lineage is most closely related to the SAR116 clade (Luo, 2015) but differs from this lineage in that it is relatively less abundant in the marine environment and its cultured representatives can display copiotrophic lifestyles. For example, bacteria of the *Rhodospirillales* genus *Thalassospira* isolated in the MS were found to degrade and display chemotaxis towards phosphonates (Hütz *et al*., 2011). In turn, C–P lyase had been previously observed to be prevalent in the *Roseobacter* clade strains with sequenced genomes (Sosa *et al*., 2017). Even though our analysis of MAGs and C–P lyase sequences revealed some of the phylogenetic diversity of bacteria containing C–P lyase, particularly among the *Alphaproteobacteria*, the identity of other clusters of C–P lyase sequences should be explored further as more genomes become available including those similar to *Actinobacteria* C–P lyase and those found in MAGs of unclassified *Gammaproteobacteria* (Supporting Information Fig. S1). In the MS, for example, phosphonate transport genes have been associated with an uncultivated group of marine *Actinobacteria* (Ghai *et al*., 2013) but representative isolates are needed to determine if this group also contains C–P lyase and if this corresponds to the *Actinobacteria* C–P lyase enriched in the MS and NAO (Fig. 4A).

The relatively high enrichment of C–P lyase in *Pelagibacter*, the SAR116 clade (> 100% or > 1 copy cell^−1^; Fig. 4) and in MAGs representative of the *Rhodospirillales* and *Rhodobacteraceae* (S2) in the EPZ in the MS and NAO, suggests that in these regions some bacteria may carry multiple copies of the C–P lyase pathway in their genome. It may also be that *phnJ* sequences classified as *Pelagibacter* or SAR116 clade C–P lyase were associated with additional groups of closely related bacteria but for which reference genomes are not available. A mechanism that would explain multiple copies of C–P lyase per cell is if bacteria encode the *phn* operon in plasmids, extrachromosomal elements of circular DNA that can replicate independently from the host genome. Plasmids may allow bacteria to regulate the copy number and expression of the C–P lyase pathway as needed to respond quickly to a shortage of Pi. The bacteria *Sinorhizobium meliloti* and *Mesorhizobium loti* of the order *Rhizobiales* of *Alphaproteobacteria* are known to carry the C–P lyase gene operon in plasmids (Huang *et al*., 2005) and several of the C–P lyase *phnJ* sequences we identified in the *Tara* Oceans metagenomes were closely related to sequences found in cultured representatives and MAGs of this taxonomic group (Supporting Information Fig. S1). Some members of the *Roseobacter* clade are also known to maintain low‐copy plasmids (~12 copies cell^−1^; Pradella *et al*., 2010) and these can carry genes that may support adaptations to their ecological niche (Petersen *et al*., 2013). Phylogenetic and genomic evidence suggests that the C–P lyase pathway has been acquired to a large degree through horizontal gene transfer, a mechanism thought to be facilitated by plasmids (Huang *et al*., 2005). Therefore, the lateral transfer of plasmids may also explain how several lineages of marine *Alphaproteobacteria* acquired the C–P lyase pathway.

### *Phosphonate cycling and oceanic aerobic methanogenesis*

The enrichment of C–P lyase in diverse bacterial populations in the MS and NAO adds further support to the hypothesis that phosphonate catabolism is an important means to obtain Pi in these regions. Acquiring C–P lyase in these Pi‐limited environments must be advantageous to bacteria because low Pi concentrations are conducive to active expression of this pathway through the Pho regulon, as observed in the Sargasso Sea for the marine filamentous cyanobacterium *Trichodesmium* (Dyhrman *et al*., 2006) and in cultures of *Pelagibacterales* sp. strain HTCC7211 (Carini *et al*., 2014), and because phosphonates present in HMW DOM could be readily used by Pi‐starved bacterial communities and bacterial isolates encoding C–P lyase (Repeta *et al*., 2016; Sosa *et al*., 2017).

Given that the occurrence of C–P lyase in *Pelagibacter* populations appears to be constrained to the MS and the NAO regions (Figs. 3C and 4; see also S2) other widespread bacterial groups containing C–P lyases such as the SAR116 clade, *Roseobacter* and *Rhodospirillales* populations may be responsible for the degradation of DOM phosphonates outside these regions. In support of this hypothesis, our analysis revealed a high relative abundance of C–P lyase in several of these lineages across all ocean regions and depths, especially among the *Rhodospirillales* (Fig. 4; see also S2). Our survey of Station ALOHA C–P lyase also identified several sequences similar to those of *Roseobacter* and *Rhodospirillales* but did not reveal matches to *Pelagibacter* (Supporting Information Fig. S1; see also the expanded tree in the Supporting Information). C–P lyase abundance at Station ALOHA averages 0.25%–0.33% (Sosa *et al*., 2017), in agreement with the estimates obtained in this study for the NPO region (Fig. 3A). The isolation of *Roseobacter* clade bacteria containing C–P lyase in this ocean region which were capable of degrading HMW DOM phosphonates (Sosa *et al*., 2017) also supports this view. These bacterial populations encoding C–P lyase are expected to acquire some cellular P from HMW DOM phosphonates.

As a result of DOM phosphonate degradation, methane (when the substrate is methylphosphonate) and other hydrocarbons are released into marine surface waters. Repeta *et al*. (2016) proposed that HMW DOM methylphosphonate degradation could explain the oceanic methane paradox, that is the slight supersaturation of methane observed in fully oxygenated marine surface waters (Kiene, 1991) which results in an outward flux of methane from the ocean to the atmosphere. The Pi‐limiting conditions and the enrichment of C–P lyase in the EPZ in the MS and the NAO suggest that the utilization of phosphonates, and perhaps the production of methane, is greater in these environments than in other ocean regions. However, a comprehensive analysis of methane dynamics across oligotrophic ocean regions with different nutrient regimes is needed to further evaluate the role of Pi in bacterial phosphonate cycling and methanogenesis.

In summary, our study provides evidence that supports the role of Pi as an important nutrient controlling the abundance and distribution of the bacterial C–P lyase pathway in the upper ocean. In addition to Pi, the availability of Fe may also shape the distribution patterns of C–P lyase. Pi concentration might also affect the aerobic oceanic methane source strength, but this connection requires further understanding of the ecology of C–P lyase and marine phosphonates as well as additional observations of methane dynamics in the ocean. The acquisition and enrichment of C–P lyase in *Pelagibacter*, the SAR116 clade, *Roseobacter* and other abundant bacterial populations in the MS and NAO may allow these abundant organisms to subsist and compete for P stored in DOM phosphonates and illustrates how the selective pressure exerted by Pi depletion has made a genomic imprint at the community level. These results warrant field studies in Pi‐limited ocean regions to further evaluate the role that Pi, Fe, and different bacterial groups encoding C–P lyase play in the degradation of phosphonates and methanogenesis.

## Experimental procedures

### *Identification and quantification of P acquisition genes*

To quantify and compare the abundance of genes involved in P acquisition functions we queried the Ocean Microbial Reference Gene Catalogue (OM‐RGC) dataset (ftp://ftp.sra.ebi.ac.uk/vol1/ERA412/ERA412970/tab/OM‐RGC_seq.release.tsv.gz; see also http://ocean-microbiome.embl.de/companion.html) from the *Tara* Oceans expedition (Sunagawa *et al*. 2015) for protein‐coding genes based on their assigned Clusters of Orthologous Groups (COG) or Kyoto Encyclopaedia of Genes and Genomes (KEGG) identifiers. These included the high‐affinity Pi transport genes *pstA* (COG0581), *pstB* (COG1117), *pstC* (COG0573) and *pstS* (COG0226); the C–P lyase pathway genes *phnG* (COG3624), *phnH* (COG3625), *phnI* (COG3626), *phnJ* (COG3627), *phnK* (COG4107) and *phnL* (COG4778); the phosphonate membrane transport proteins encoded by *phnC* (COG3638), *phnD* (COG3221) and *phnE* (COG2629); the phosphonate degradation genes *phnW* (K03430), which encodes 2‐aminoethylphosphonate pyruvate transaminase; *phnX* (K05306), which encodes phosphonoacetaldehyde hydrolase; and *phnA* (COG1524), which encodes phosphonoacetate hydrolase; and the APases encoded by *phoA* (COG1785), *phoD* (COG3540) and *phoX* (COG3211). In the comparison, we included the Pi starvation response regulator encoded by *phoB* (K07657) and the phospholipase C involved in phospholipid substitution encoded by *plcP* to serve as markers for Pi deficiency. Homologues of *plcP* were identified in the OM‐RGC by similarity with the protein sequence of *Phaeobacter* sp. MED139 using TBLASTN with an *e*‐value cutoff of 10^−40^ as described by Sebastián *et al*. (2016). We extracted the abundance of each gene from the *Tara* Oceans OM‐RGC profiles dataset (http://ocean-microbiome.embl.de/data/TARA243.gene.profile.release.gz) which was calculated from read counts mapped to each reference gene normalized by the gene‐length (Sunagawa *et al*., 2015). The total abundance of OM‐RGC sequences matching the same reference gene function was normalized to the total abundance of OM‐RGC sequences corresponding to the single‐copy marker gene *recA* (COG0468) as performed in previous studies (Martinez *et al*., 2010). This enabled a comparison of the relative abundance of P acquisition pathways on a per‐genome level across the *Tara* Oceans metagenomes. For the present study, we focused on metagenomes obtained from samples collected on 0.22 μm pore‐size membranes and pre‐filtered by a 0.45, 1.6 or 3.0 μm pore size membrane. With respect to depth, samples spanned the epipelagic zone (EPZ), which included surface waters (5 m) and the DCM layer (17–183 m), as well as the mesopelagic zone (MPZ), which extended from 200 m to 1000 m. We also compared the abundance of P acquisition genes across representative ocean regions based on the biogeographic classification of the sampling location of each metagenome: Mediterranean Sea (MS), Red Sea (RS), North Atlantic Ocean (NAO), South Atlantic Ocean (SAO), North Pacific Ocean (NPO), South Pacific Ocean (SPO), Indian Ocean (IO) and Southern Ocean (SO).

### *Statistical analysis*

We used the non‐parametric Kruskal–Wallis test to evaluate the significance level of differences in relative gene abundance between ocean regions and applied the Wilcoxon test with the Benjamini–Hochberg correction for multiple testing to calculate pairwise comparisons between ocean regions. Significance tests were implemented with the *stats* R software package (R Core Team, 2018). To evaluate which environmental parameters could have an effect on the distribution of P acquisition genes we computed Pearson correlations using the *Hmisc* R software package (Harrel *et al*., 2018).

### *Environmental variables*

The environmental parameters tested included sampling depth, temperature, dissolved oxygen, silicate, inorganic nitrogen (N; includes nitrate plus nitrite) and the N to Pi (N:P) molar concentration ratio from *Tara* Oceans. We also included in the analysis environmental parameters from the Pelagic Interactions Scheme for Carbon and Ecosystem Studies (PISCES) v3 ocean biogeochemical model (Aumont *et al*., 2015) and from the World Ocean Atlas (WOA) 2009 (Garcia *et al*., 2010). From PISCES, we computed the monthly climatology of dissolved iron (Fe), silicate, nitrate, Pi, chlorophyll, oxygen and phytoplankton concentration matching the *Tara* Oceans metagenomes sampling locations with a spatial tolerance of 0.3° and a depth tolerance of 5 m. PISCES data were extracted using the Opedia software and database (Ashkesari and Berthiaume, 2018). From the WOA mean annual climatology, we obtained Pi and nitrate concentrations matching *Tara* Oceans sampling locations within one degree of latitude and longitude and no more than 5 m depth for samples from the EPZ. *Tara* Oceans sampling stations were matched to WOA stations using the nearest neighbour search function in the *RANN* package for R (Arya *et al*., 2019). The WOA and PISCES data provided two additional independent datasets to evaluate the correlation of environmental parameters with gene abundance.

### *Regression models of the response of P acquisition gene abundance to phosphate*

To further describe the response of P acquisition genes to Pi, we fitted linear regression models of the log‐transformed relative abundance of each reference gene normalized to the log‐transformed mean annual Pi concentration obtained from the WOA at the corresponding sampling location and depth of each *Tara* Oceans metagenome. We also fitted a linear regression model of the log‐transformed gene relative abundance with two predictors, ocean region and log‐transformed Pi concentration from WOA, and evaluated the significance of each predictor using the ANOVA function in the *car* R package. To compare the slopes of the regression lines, we computed the z statistic as the difference between the two slopes divided by the standard error of the difference between the slopes and evaluated its significance at the 0.05 level.

### *Phylogenetic analysis of C–P lyase*

The OM‐RGC includes a taxonomic profile of its genes from the phylum to the species level. However, because many OM‐RGC sequences lack classification at various taxonomic levels we performed a phylogenetic analysis of the C–P lyase pathway protein PhnJ. We downloaded 201 reference C–P lyase (PhnJ) protein sequences available from eggNOG v4.5.1 (Huerta‐Cepas *et al*., 2016) and five additional reference sequences obtained from NCBI belonging to *Actinobacteria* genomes, and aligned them with C–P lyase sequences from the OM‐RGC and from the Station ALOHA gene catalogue (Mende *et al*., 2017). C–P lyase sequences in the Station ALOHA gene catalogue were identified by a BLASTN search using the OM‐RGC *phnJ* nucleotide sequences as queries. We also included in the analysis C–P lyase sequences identified in metagenome‐assembled genomes (MAGs) from *Tara* Oceans (Tully *et al*., 2018) to aid with the classification of OM‐RGC C–P lyase sequences. The classification of MAGs was based on a phylogenetic analysis of several single‐copy marker genes and is described in detail in Tully *et al*. (2018). To identify *phnJ* genes in these MAGs, the protein‐coding regions in the assemblies available under NCBI BioProject PRJNA391943 were downloaded and subject to a BLASTP search using the translated sequence of *phnJ* genes identified in the OM‐RGC. The BLASTP search provided 122 additional PhnJ reference sequences from these MAGs. A multiple sequence alignment of PhnJ was constructed using Clustal Omega with mBed‐like clustering enabled (Sievers *et al*., 2011). Alignment columns with >10% gaps were removed and only unique sequences were kept. The final alignment consisted of 440 unique sequences with 276 positions. The complete set of OM‐RGC and reference PhnJ sequences and the amino acid sequence alignment without trimming are available in fasta format in the Supporting Information. We then constructed a maximum likelihood tree with RAxML v8 (Stamatakis, 2014) using the Le and Gascuel (2008) amino acid substitution matrix, the gamma model of rate heterogeneity, rapid bootstrapping (500 iterations) and maximum likelihood searches. RAxML was implemented on the Cyberinfrastructure for Phylogenetic Research (CIPRES) Science Gateway (Miller *et al*., 2010). A conservative taxonomic classification was assigned to each OM‐RGC sequence based on their placement in the phylogenetic tree and on the taxonomic identity of the most closely related sequence in a reference genome or MAG. An expanded version of the phylogenetic tree of PhnJ sequences can be accessed through the interactive tree of life (iTOL) website (see Supporting Information). To estimate the relative contribution of different bacterial groups to the total C–P lyase abundance in a metagenomic sample, OM‐RGC *phnJ* sequences with the same taxonomic classification were grouped and their abundances summed and normalized by the total abundance of *phnJ*.

### *Taxon‐specific enrichment of C–P lyase*

To calculate the percentage of organisms containing C–P lyase within a taxonomic group in each *Tara* Oceans sample, *phnJ* genes were binned based on their amino acid sequence phylogeny and their abundances summed and normalized by the mean abundance of 40 single‐copy marker genes matching each taxon bin based on the OM‐RGC classification (Sunagawa *et al*., 2015). Single‐copy marker genes were identified in the OM‐RGC with the program fetchMG (Sunagawa *et al*., 2013) and their abundance extracted from the OM‐RGC profiles dataset.

To further resolve the abundance of C–P lyase in specific populations of organisms at a finer taxonomic level, the raw reads of each *Tara* Oceans metagenome of the 0.22 μm size‐fraction were mapped to MAG assemblies (Tully *et al*., 2018) possessing C–P lyase and to the genome of *Pelagibacterales* sp. strain HTCC7211 (GenBank accession ABVS01000001) which also contains the C–P lyase pathway (Carini *et al*., 2014). *Tara* Oceans metagenomic reads were obtained from the European Molecular Biology Laboratory‐European Bioinformatics Institute and MAG assemblies (Tully *et al*., 2018) were downloaded from https://ndownloader.figshare.com/files/8849371. Reads were mapped against the genome assemblies using Bowtie 2 v2.2.4 (Langmead and Salzberg, 2012) and coding DNA sequences were identified with Prodigal v2.6.2 (Hyatt *et al*., 2010). To calculate the coverage of each gene in the assemblies, we employed the Bedtools suite (v2.27.1) coverage tool (Quinlan and Hall, 2010). For each metagenomic sample, the relative abundance of each MAG was calculated by normalizing the coverage of *phnJ* by the mean coverage of 40 single‐copy marker genes (identified with fetchMG) and was expressed as a percentage of organisms with C–P lyase.

## Conflict of interest

The authors declare that they have no conflict of interest.

## Supporting information


**Table S1.** Linear regression models between gene relative abundance and mean annual phosphate concentration data from the World Ocean Atlas.
**Figure S1.** Maximum likelihood tree of C‐P lyase (PhnJ) protein sequences retrieved from the Ocean Microbial Reference Gene Catalog (OM‐RGC)
**Figure S2.** Taxon‐specific enrichment of C‐P lyase across depth zones and ocean regions.
**Figure S3.** C‐P lyase pathway phn gene operons from representative Tara Oceans metagenome assembled‐genomes (MAGs).

**Appendix S1.** *Tara* Oceans OM‐RGC and reference sequences of C‐P lyase protein PhnJ.

**Appendix S2.** Clustal Omega alignment of C‐P lyase protein PhnJ.

## References

[emi14628-bib-0001] Ammerman, J.W. , Hood, R.R. , Case, D.A. , and Cotner, J.B. (2003) Phosphorus deficiency in the Atlantic: an emerging paradigm in oceanography. EOS Trans Am Geophys Union 84: 165–170.

[emi14628-bib-0002] Arya, S. , Mount, D. , Kemp, S.E. , and Jefferis, G. (2019) *RANN: Fast Nearest Neighbour Search (Wraps ANN Library) Using L2 Metric. R package version 2.6.1* URL https://cran.r-project.org/package=RANN.

[emi14628-bib-0074] Ashkezari, M.D. , and Berthiaume, C. (2018) Opedia: Space‐Time Compliance (version 0.1.39). Zenodo. 10.5281/zenodo.1440319.

[emi14628-bib-0003] Aumont, O. , Ethé, C. , Tagliabue, A. , Bopp, L. , and Gehlen, M. (2015) PISCES‐v2: an ocean biogeochemical model for carbon and ecosystem studies. Geosci Model Dev 8: 2465–2513.

[emi14628-bib-0004] Björkman, K. , and Karl, D. (2003) Bioavailability of dissolved organic phosphorus in the euphotic zone at Station ALOHA, North Pacific Subtropical Gyre. Limnol Oceanogr 48: 1049–1057.

[emi14628-bib-0005] Carini, P. , White, A.E. , Campbell, E.O. , and Giovannoni, S.J. (2014) Methane production by phosphate‐starved SAR11 chemoheterotrophic marine bacteria. Nat Commun 5: 4346. 25000228 10.1038/ncomms5346

[emi14628-bib-0006] Carini, P. , Van Mooy, B.A.S. , Thrash, J.C. , White, A. , Zhao, Y. , Campbell, E.O. , *et al* (2015) SAR11 lipid renovation in response to phosphate starvation. Proc Natl Acad Sci USA 112: 7767–7772. 26056292 10.1073/pnas.1505034112 PMC4485111

[emi14628-bib-0007] Cavendar‐Bares, K.K. , Karl, D.M. , and Chisholm, S.W. (2001) Nutrient gradients in the western North Atlantic Ocean: relationship to microbial community structure and comparison to pattern in the Pacific Ocean. Deep Sea Res Part I Oceanogr Res Pap 48: 2373–2395.

[emi14628-bib-0008] Chen, C.‐M. , Ye, Q.‐Z. , Zhu, Z. , Wanner, B.L. , and Walsh, C.T. (1990) Molecular biology of carbon‐phosphorus bond cleavage. Cloning and sequencing of the *phn* (*psiD*) genes involved in alkylphosphonate uptake and C‐P lyase activity in *Escherichia coli* B. J Biol Chem 265: 4461–4471. 2155230

[emi14628-bib-0009] Chin, J.P. , Quinn, J.P. , and McGrath, J.W. (2018) Phosphate insensitive aminophosphonate mineralisation within oceanic nutrient cycles. ISME J 12: 973–980. 29339823 10.1038/s41396-017-0031-7 PMC5864244

[emi14628-bib-0010] Coleman, M.L. , and Chisholm, S.W. (2010) Ecosystem‐specific selection pressures revealed through comparative population genomics. Proc Natl Acad Sci USA 107: 18634–18639. 20937887 10.1073/pnas.1009480107 PMC2972931

[emi14628-bib-0011] Cotner, J.B. , Ammerman, J.W. , Peele, E.R. , and Bentzen, E. (1997) Phosphorus‐limited bacterioplankton growth in the Sargasso Sea. Aquat Microb Ecol 13: 141–149.

[emi14628-bib-0012] Dyhrman, S.T. , Chappell, P.D. , Haley, S.T. , Moffett, J.W. , Orchard, E.D. , Waterbury, J.B. , and Webb, E.A. (2006) Phosphonate utilization by the globally important marine diazotroph *Trichodesmium* . Nature 439: 68–71. 16397497 10.1038/nature04203

[emi14628-bib-0013] Dyhrman, S. , Ammerman, J. , and Van Mooy, B. (2007) Microbes and the marine phosphorus cycle. Oceanography 20: 110–116.

[emi14628-bib-0014] Feingersch, R. , Suzuki, M.T. , Shmoish, M. , Sharon, I. , Sabehi, G. , Partensky, F. , and Béjà, O. (2010) Microbial community genomics in eastern Mediterranean Sea surface waters. ISME J 4: 78–87. 19693100 10.1038/ismej.2009.92

[emi14628-bib-0015] Garcia, H.E. , Locarnini, R.A. , Boyer, T.P. , Antonov, J.I. , Zweng, M.M. , Baranova, O.K. , and Johnson, D.R. (2010) World Ocean atlas 2009, volume 4: nutrients (phosphate, nitrate, and silicate). NOAA World Ocean Atlas 71: 398.

[emi14628-bib-0016] Ghai, R. , Mizuno, C.M. , Picazo, A. , Camacho, A. , and Rodriguez‐Valera, F. (2013) Metagenomics uncovers a new group of low GC and ultra‐small marine Actinobacteria. Sci Rep 3: 2471. 23959135 10.1038/srep02471 PMC3747508

[emi14628-bib-0017] Guieu, C. , Bozec, Y. , Blain, S. , Ridame, C. , Sarthou, G. , and Leblond, N. (2002) Impact of high Saharan dust inputs on dissolved iron concentrations in the Mediterranean Sea. Geophys Res Lett 29: 17‐1–17‐4.

[emi14628-bib-0018] Harrell, F.E., Jr , with contributions from Charles Dupont and many others . (2018) *Hmisc: Harrell Miscellaneous. R package version 4.1‐1* URL https://cran.r-project.org/package=Hmisc.

[emi14628-bib-0019] Huang, J. , Su, Z. , and Xu, Y. (2005) The evolution of microbial phosphonate degradative pathways. J Mol Evol 61: 682–690. 16245012 10.1007/s00239-004-0349-4

[emi14628-bib-0020] Huerta‐Cepas, J. , Szklarczyk, D. , Forslund, K. , Cook, H. , Heller, D. , Walter, M.C. , *et al* (2016) eggNOG 4.5: a hierarchical orthology framework with improved functional annotations for eukaryotic, prokaryotic and viral sequences. Nucleic Acids Res 44: D286–D293. 26582926 10.1093/nar/gkv1248 PMC4702882

[emi14628-bib-0021] Hütz, A. , Schubert, K. , and Overmann, J. (2011) *Thalassospira* sp. isolated from the oligotrophic eastern Mediterranean Sea exhibits chemotaxis toward inorganic phosphate during starvation. Appl Environ Microbiol 77: 4412–4421. 21602377 10.1128/AEM.00490-11 PMC3127729

[emi14628-bib-0022] Hyatt, D. , Chen, G.‐L. , Locascio, P.F. , Land, M.L. , Larimer, F.W. , and Hauser, L.J. (2010) Prodigal: prokaryotic gene recognition and translation initiation site identification. BMC Bioinformatics 11: 119. 20211023 10.1186/1471-2105-11-119 PMC2848648

[emi14628-bib-0023] Kamat, S.S. , Williams, H.J. , Dangott, L.J. , Chakrabarti, M. , and Raushel, F.M. (2013) The catalytic mechanism for aerobic formation of methane by bacteria. Nature 497: 132–136. 23615610 10.1038/nature12061

[emi14628-bib-0024] Karl, D.M. , Björkman, K.M. , Dore, J.E. , Fujieki, L. , Hebel, D.V. , Houlihan, T. , *et al* (2001) Ecological nitrogen‐to‐phosphorus stoichiometry at station ALOHA. Deep‐Sea Res Part II Top Stud Oceanogr 48: 1529–1566.

[emi14628-bib-0025] Karl, D.M. , Beversdorf, L. , Björkman, K.M. , Church, M.J. , Martinez, A. , and DeLong, E.F. (2008) Aerobic production of methane in the sea. Nat Geosci 1: 473–478.

[emi14628-bib-0026] Kathuria, S. , and Martiny, A.C. (2010) Prevalence of a calcium‐based alkaline phosphatase associated with the marine cyanobacterium *Prochlorococcus* and other ocean bacteria. Environ Microbiol 13: 74–83. 10.1111/j.1462-2920.2010.02310.x 20649645

[emi14628-bib-0027] Kiene, R.P. (1991) Production and consumption of methane in aquatic systems In Microbial Production and Comsumption of Greenhouse Gases: Methane, Nitrogen Oxides and Halomethanes, Rogers J.E., and Whitman W.B. (eds). Washington, DC: American Society for Microbiology, pp. 111–146.

[emi14628-bib-0028] Kolowith, L.C. , Ingall, E.D. , and Benner, R. (2001) Composition and cycling of marine organic phosphorus. Limnol Oceanogr 46: 309–320.

[emi14628-bib-0029] Kononova, S.V. , and Nesmeyanova, M.A. (2002) Phosphonates and their degradation by microorganisms. Biochemistry 67: 184–195. 11952414 10.1023/a:1014409929875

[emi14628-bib-0030] Krom, M.D. , Kress, N. , Brenner, S. , and Gordon, L.I. (1991) Phosphorus limitation of primary productivity in the eastern Mediterranean Sea. Limnol Oceanogr 36: 424–432.

[emi14628-bib-0031] Krom, M.D. , Emeis, K.‐C. , and Van Cappellen, P. (2010) Why is the eastern Mediterranean phosphorus limited? Prog Oceanogr 85: 236–244.

[emi14628-bib-0032] del Valle, D.A. , and Karl, D.M. (2014) Aerobic production of methane from dissolved water‐column methylphosphonate and sinking particles in the North Pacific subtropical gyre. Aquat Microb Ecol 73: 93–105.

[emi14628-bib-0033] Langmead, B. , and Salzberg, S.L. (2012) Fast gapped‐read alignment with bowtie 2. Nat Methods 9: 357–359. 22388286 10.1038/nmeth.1923 PMC3322381

[emi14628-bib-0075] Le, S. , and Gascuel, O. (2008) An improved general amino acid replacement matrix. Mol Biol Evol 25: 1307–1320. 18367465 10.1093/molbev/msn067

[emi14628-bib-0034] Lomas, M.W. , Burke, A.L. , Lomas, D.A. , Bell, D.W. , Shen, C. , Dyhrman, S.T. , and Ammerman, J.W. (2010) Sargasso Sea phosphorus biogeochemistry: an important role for dissolved organic phosphorus (DOP). Biogeosciences 7: 695–710.

[emi14628-bib-0035] Luo, H. (2015) Evolutionary origin of a streamlined marine bacterioplankton lineage. ISME J 9: 1423–1433. 25431989 10.1038/ismej.2014.227 PMC4438329

[emi14628-bib-0036] Luo, H. , Zhang, H. , Long, R.A. , and Benner, R. (2011) Depth distributions of alkaline phosphatase and phosphonate utilization genes in the North Pacific subtropical gyre. Aquat Microb Ecol 62: 61–69.

[emi14628-bib-0037] Martinez, A. , Tyson, G.W. , and DeLong, E.F. (2010) Widespread known and novel phosphonate utilization pathways in marine bacteria revealed by functional screening and metagenomic analyses. Environ Microbiol 12: 222–238. 19788654 10.1111/j.1462-2920.2009.02062.x

[emi14628-bib-0038] Mende, D.R. , Bryant, J.A. , Aylward, F.O. , Eppley, J.M. , Nielsen, T. , Karl, D.M. , and DeLong, E.F. (2017) Environmental drivers of a microbial genomic transition zone in the ocean's interior. Nat Microbiol 2: 1367–1373. 28808230 10.1038/s41564-017-0008-3

[emi14628-bib-0039] Merchant, S.S. , and Helmann, J.D. (2012) Elemental economy: microbial strategies for optimizing growth in the face of nutrient limitation. Adv Microb Physiol 60: 91–210. 22633059 10.1016/B978-0-12-398264-3.00002-4 PMC4100946

[emi14628-bib-0040] Metcalf, W.W. , and Wanner, B.L. (1993) Mutational analysis of an *Escherichia coli* fourteen‐gene operon for phosphonate degradation, using Tn*phoA*’ elements. J Bacteriol 175: 3430–3442. 8388873 10.1128/jb.175.11.3430-3442.1993 PMC204742

[emi14628-bib-0041] Miller, M.A , Pfeiffer, W. , and Schwartz, T. (2010) *Creating the CIPRES Science Gateway for inference of large phylogenetic trees*. 2010 Gateway Computing Environments Workshop (GCE), New Orleans, LA, pp. 1–8.

[emi14628-bib-0042] Moore, C.M. , Mills, M.M. , Arrigo, K.R. , Berman‐Frank, I. , Bopp, L. , Boyd, P.W. , *et al* (2013) Processes and patterns of oceanic nutrient limitation. Nat Geosci 6: 701–710.

[emi14628-bib-0043] Oh, H.M. , Kwon, K.K. , Kang, I. , Kang, S.G. , Lee, J.H. , Kim, S.J. , and Cho, J.C. (2010) Complete genome sequence of “*Candidatus* Puniceispirillum marinum” IMCC1322, a representative of the SAR116 clade in the *Alphaproteobacteria* . J Bacteriol 192: 3240–3241. 20382761 10.1128/JB.00347-10 PMC2901696

[emi14628-bib-0044] Petersen, J. , Frank, O. , Göker, M. , and Pradella, S. (2013) Extrachromosomal, extraordinary and essential—the plasmids of the *Roseobacter* clade. Appl Microbiol Biotechnol 97: 2805–2815. 23435940 10.1007/s00253-013-4746-8

[emi14628-bib-0045] Powley, H.R. , Krom, M.D. , and Van Cappellen, P. (2017) Understanding the unique biogeochemistry of the Mediterranean Sea: insights from a coupled phosphorus and nitrogen model. Global Biogeochem Cycles 31: 1010–1031.

[emi14628-bib-0046] Pradella, S. , Päuker, O. , and Petersen, J. (2010) Genome organisation of the marine *Roseobacter* clade member *Marinovum algicola* . Arch Microbiol 192: 115–126. 20039020 10.1007/s00203-009-0535-2

[emi14628-bib-0047] Quinlan, A.R. , and Hall, I.M. (2010) BEDTools: a flexible suite of utilities for comparing genomic features. Bioinformatics 26: 841–842. 20110278 10.1093/bioinformatics/btq033 PMC2832824

[emi14628-bib-0048] Quinn, J.P. , Kulakova, A.N. , Cooley, N.A. , and McGrath, J.W. (2007) New ways to break an old bond: the bacterial carbon‐phosphorus hydrolases and their role in biogeochemical phosphorus cycling. Environ Microbiol 9: 2392–2400. 17803765 10.1111/j.1462-2920.2007.01397.x

[emi14628-bib-0049] R Core Team . (2018) R: A Language and Environment for Statistical Computing. Vienna, Austria: R Foundation for Statistical Computing URL https://www.r-project.org/.

[emi14628-bib-0050] Repeta, D.J. , Ferrón, S. , Sosa, O.A. , Johnson, C.G. , Repeta, L.D. , Acker, M. , *et al* (2016) Marine methane paradox explained by bacterial degradation of dissolved organic matter. Nat Geosci 9: 884–887.

[emi14628-bib-0051] Sebastián, M. , and Ammerman, J.W. (2009) The alkaline phosphatase PhoX is more widely distributed in marine bacteria than the classical PhoA. ISME J 3: 563–572. 19212430 10.1038/ismej.2009.10

[emi14628-bib-0052] Sebastián, M. , Smith, A.F. , González, J.M. , Fredricks, H.F. , Van Mooy, B. , Koblížek, M. , *et al* (2016) Lipid remodelling is a widespread strategy in marine heterotrophic bacteria upon phosphorus deficiency. ISME J 10: 968–978. 26565724 10.1038/ismej.2015.172 PMC4796936

[emi14628-bib-0053] Seweryn, P. , Van, L.B. , Kjeldgaard, M. , Russo, C.J. , Passmore, L.A. , Hove‐Jensen, B. , *et al* (2015) Structural insights into the bacterial carbon‐phosphorus lyase machinery. Nature 525: 68–72. 26280334 10.1038/nature14683 PMC4617613

[emi14628-bib-0054] Sievers, F. , Wilm, A. , Dineen, D. , Gibson, T.J. , Karplus, K. , Li, W. , *et al* (2011) Fast, scalable generation of high‐quality protein multiple sequence alignments using Clustal omega. Mol Syst Biol 7: 539. 21988835 10.1038/msb.2011.75 PMC3261699

[emi14628-bib-0055] Singh, A. , Lomas, M.W. , and Bates, N.R. (2013) Revisiting N_2_ fixation in the North Atlantic Ocean: significance of deviations from the Redfield ratio, atmospheric deposition and climate variability. Deep Sea Res Part II Top Stud Oceanogr 93: 148–158.

[emi14628-bib-0056] Sosa, O.A. , Repeta, D.J. , Ferrón, S. , Bryant, J.A. , Mende, D.R. , Karl, D.M. , and DeLong, E.F. (2017) Isolation and characterization of bacteria that degrade phosphonates in marine dissolved organic matter. Front Microbiol 8: 1786. 29085339 10.3389/fmicb.2017.01786 PMC5649143

[emi14628-bib-0057] Stamatakis, A. (2014) RAxML version 8: a tool for phylogenetic analysis and post‐analysis of large phylogenies. Bioinformatics 30: 1312–1313. 24451623 10.1093/bioinformatics/btu033 PMC3998144

[emi14628-bib-0058] Sunagawa, S. , Mende, D.R. , Zeller, G. , Izquierdo‐Carrasco, F. , Berger, S.A. , Kultima, J.R. , *et al* (2013) Metagenomic species profiling using universal phylogenetic marker genes. Nat Methods 10: 1196–1199. 24141494 10.1038/nmeth.2693

[emi14628-bib-0059] Sunagawa, S. , Coelho, L.P. , Chaffron, S. , Kultima, J.R. , Labadie, K. , Salazar, G. , *et al* (2015) Structure and function of the global ocean microbiome. Science 348: 1261359. 25999513 10.1126/science.1261359

[emi14628-bib-0060] Thingstad, T.F. , and Rassoulzadegan, F. (1995) Nutrient limitations, microbial food webs, and ‘biological C‐pumps’: suggested interactions in a P‐limited Mediterranean. Mar Ecol Prog Ser 117: 299–306.

[emi14628-bib-0061] Thingstad, T.F. , Krom, M.D. , Mantoura, R.F.C. , Flaten, G.A.F. , Groom, S. , Herut, B. , *et al* (2005) Nature of phosphorus limitation in the ultraoligotrophic eastern Mediterranean. Science 309: 1068–1071. 16099984 10.1126/science.1112632

[emi14628-bib-0062] Torres‐Valdés, S. , Roussenov, V.M. , Sanders, R. , Reynolds, S. , Pan, X. , Mather, R. , *et al* (2009) Distribution of dissolved organic nutrients and their effect on export production over the Atlantic Ocean. Global Biogeochem Cycles 23: GB4019.

[emi14628-bib-0063] Tully, B.J. , Graham, E.D. , and Heidelberg, J.F. (2018) The reconstruction of 2,631 draft metagenome‐assembled genomes from the global oceans. Sci Data 5: 170203. 29337314 10.1038/sdata.2017.203 PMC5769542

[emi14628-bib-0064] Van Mooy, B.A.S. , Rocap, G. , Fredricks, H.F. , Evans, C.T. , and Devol, A.H. (2006) Sulfolipids dramatically decrease phosphorus demand by picocyanobacteria in oligotrophic marine environments. Proc Natl Acad Sci USA 103: 8607–8612. 16731626 10.1073/pnas.0600540103 PMC1482627

[emi14628-bib-0065] Van Mooy, B.A.S. , Fredricks, H.F. , Pedler, B.E. , Dyhrman, S.T. , Karl, D.M. , Koblížek, M. , *et al* (2009) Phytoplankton in the ocean use non‐phosphorus lipids in response to phosphorus scarcity. Nature 458: 69–72. 19182781 10.1038/nature07659

[emi14628-bib-0066] Villarreal‐Chiu, J.F. , Quinn, J.P. , and McGrath, J.W. (2012) The genes and enzymes of phosphonate metabolism by bacteria, and their distribution in the marine environment. Front Microbiol 3: 19. 22303297 10.3389/fmicb.2012.00019 PMC3266647

[emi14628-bib-0067] Wackett, L.P. , Wanner, B.L. , Venditti, C.P. , and Walsh, C.T. (1987) Involvement of the phosphate regulon and the *psiD* locus in carbon‐phosphorus lyase activity of *Escherichia coli* K‐12. J Bacteriol 169: 1753–1756. 3549702 10.1128/jb.169.4.1753-1756.1987 PMC212012

[emi14628-bib-0068] Wanner, B.L. (1998) Phosphate signaling and the control of gene expression in *Escherichia coli* In Metal Ions in Gene Regulation, Silver S., and Walden W. (eds). Boston, MA: Springer, pp. 104–128.

[emi14628-bib-0069] Wanner, B.L. , and Chang, B.D. (1987) The *phoBR* operon in *Escherichia coli* K‐12. J Bacteriol 169: 5569–5574. 2824439 10.1128/jb.169.12.5569-5574.1987 PMC213987

[emi14628-bib-0070] White, A.K. , and Metcalf, W.W. (2007) Microbial metabolism of reduced phosphorus compounds. Annu Rev Microbiol 61: 379–400. 18035609 10.1146/annurev.micro.61.080706.093357

[emi14628-bib-0071] Wu, J. , Sunda, W.G. , Boyle, E.A. , and Karl, D.M. (2000) Phosphate depletion in the western North Atlantic Ocean. Science 289: 759–762. 10926534 10.1126/science.289.5480.759

[emi14628-bib-0072] Young, C.L. , and Ingall, E. (2010) Marine dissolved organic phosphorus composition: insights from samples recovered using combined electrodialysis/reverse osmosis. Aquat Geochem 16: 563–574.

[emi14628-bib-0073] Zaccone, R. , Boldrin, A. , Caruso, G. , la Ferla, R. , Maimone, G. , Santinelli, C. , and Turchetto, M. (2012) Enzymatic activities and prokaryotic abundance in relation to organic matter along a West‐East Mediterranean transect (TRANSMED cruise). Microb Ecol 64: 54–66. 22349935 10.1007/s00248-012-0011-4

