# Supplementary material for: Phosphate‐limited ocean regions select for bacterial populations enriched in the carbon–phosphorus lyase pathway for phosphonate degradation
Source: Environ Microbiol. 2019 May 27;21(7):2402–14. doi: 10.1111/1462-2920.14628 (PMC6852614; doi:10.1111/1462-2920.14628)
Supplement: Supplementary file 1 — Table S1. Linear regression models between gene relative abundance and mean annual phosphate concentration data from the World Ocean Atlas. Figure S1. Maximum likelihood tree of C‐P lyase (PhnJ) protein sequences retrieved from the Ocean Microbial Reference Gene Catalog (OM‐RGC) Figure S2. Taxon‐specific enrichment of C‐P lyase across depth zones and ocean regions. Figure S3. C‐P lyase pathway phn gene operons from representative Tara Oceans metagenome assembled‐genomes (MAGs). [file EMI-21-2402-s001.pdf]

Supporting Information

Table S1

Figure S1

Figure S2

Figure S3

References

Phosphate-limited ocean regions select for bacterial populations enriched in the carbon-phosphorus lyase pathway for phosphonate degradation

Oscar A. Sosa[1*†], Daniel J. Repeta[2], Edward F. DeLong[1], Mohammad D. Ashkezari[3], and David M. Karl[1]

[1]Daniel K. Inouye Center for Microbial Oceanography: Research and Education, University of Hawai‘i at Mānoa, Honolulu, HI 96822, USA

[2]Department of Marine Chemistry and Geochemistry, Woods Hole Oceanographic Institution, Woods Hole, MA 02540, USA

[3]School of Oceanography, University of Washington, Seattle, WA 98105, USA

*To whom correspondence should be addressed

Tel: (+1) 808 956 8779

E-mail: ososa@hawaii.edu

[†]Present address: Department of Biology, University of Puget Sound, Tacoma, WA 98416, USA.

E-mail: ososa@pugetsound.edu

Table S1. Linear regression models between gene relative abundance and mean annual phosphate concentration data from the World Ocean Atlas.

Gene	COG/KEGG	Intercept	Slope	Slope S.E.	r^2	*F*	*p**
pstC	COG0573	-2.69	-0.80	0.06	0.69	202 (1,90)	***
pstA	COG0581	-2.77	-0.81	0.06	0.67	186 (1,90)	***
pstB	COG1117	-2.53	-0.72	0.05	0.67	186 (1,90)	***
pstS	COG0226	-1.95	-0.67	0.05	0.64	159 (1,90)	***
phoB	K07657	-1.96	-0.47	0.05	0.52	101 (1,90)	***
plcP [#]	K01175	-4.53	-0.89	0.10	0.45	75 (1,90)	***
phnH	COG3625	-7.96	-1.30	0.16	0.42	66 (1,90)	***
phnK	COG4107	-7.74	-1.24	0.16	0.40	62 (1,90)	***
phnG	COG3624	-7.65	-1.14	0.15	0.39	59 (1,90)	***
phnI	COG3626	-7.34	-1.14	0.15	0.39	59 (1,90)	***
phnJ	COG3627	-7.49	-1.13	0.16	0.35	50 (1,90)	***
phnC	COG3638	-2.19	-0.28	0.04	0.35	50 (1,90)	***
phnL	COG4778	-7.33	-1.05	0.16	0.33	45 (1,90)	***
phnD	COG3221	-1.95	-0.23	0.04	0.28	37 (1,90)	***
phoX	COG3211	-3.52	-0.38	0.07	0.23	28 (1,90)	***
phnE	COG2629	-1.89	-0.18	0.05	0.10	11 (1,90)	**
phnA	COG1524	-1.60	0.12	0.04	0.10	11 (1,90)	**
phnX	K05306	-5.73	-0.40	0.13	0.08	9 (1,90)	**
phoA	COG1785	-2.52	0.14	0.06	0.05	6 (1,90)	*
phoD	COG3540	-2.19	-0.06	0.05	0.00	1 (1,90)	0.24
phnW	K03430	-3.11	-0.02	0.05	-0.01	0 (1,90)	0.73

* Significance *p* value of linear model: *** <0.001, ** <0.01, * <0.05

[#] *plcP* homologs were identified by BLAST analysis. See experimental procedures.

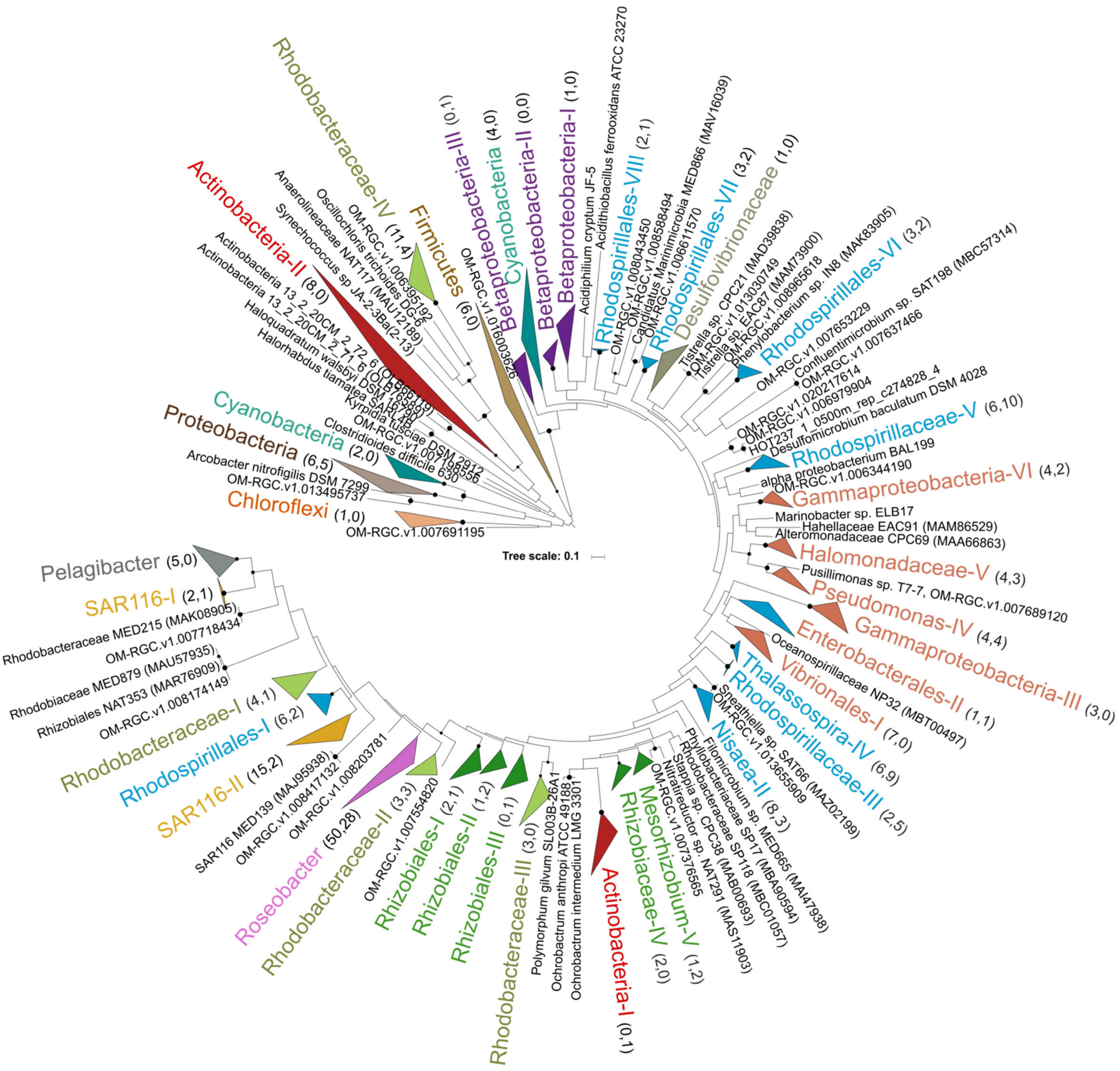

Figure S1. Maximum likelihood tree of C-P lyase (PhnJ) protein sequences retrieved from the Ocean Microbial Reference Gene Catalog (OM-RGC). Bootstrap values are represented by the circle size and range from 70-100%. The tree scale represents number of substitutions per site. The tree also contains references sequences from NCBI and EggNOG. The protein accession numbers of PhnJ sequences retrieved from MAGs are indicated in parentheses in the corresponding tree leaf. For simplicity, nodes were collapsed and color coded to indicate the predominant taxonomic affiliation of the sequences therein. The Roman numeral assigned to each collapsed node clade name can be cross referenced with Fig. S2 describing the relative occurrence of C-P lyase in MAGs. The number of OM-RGC PhnJ sequences and *Tara* Oceans MAGs containing C-P lyase that clustered with each collapsed node are indicated in parenthesis (#OM-GC PhnJ, #MAGs with C-P lyase). A fully expanded tree is available through iTOL (https://itol.embl.de/tree/12817145340278153895872).

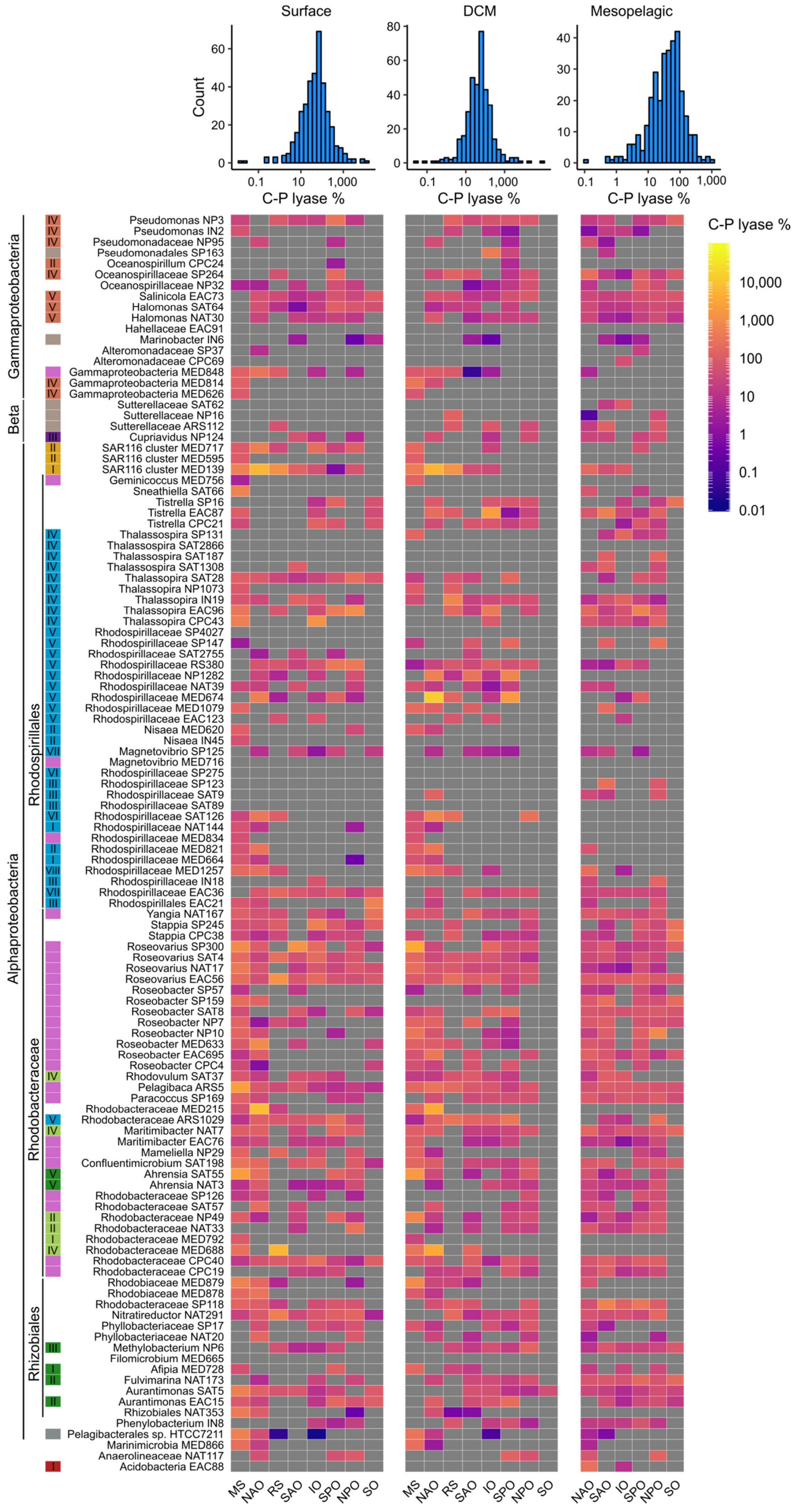

Surface
DCM
Mesopelagic
Count
C-P lyase %
C-P lyase %
10,000
1,000
100
10
1
0.1
0.01
Gammaproteobacteria
Beta
Alphaproteobacteria
Rhodospirillales
Rhodobacteraceae
Rhizobiales
Pseudomonas NP3
Pseudomonas IN2
Pseudomonadaceae NP95
Pseudomonadales SP163
Oceanospirillum CPC24
Oceanospirillaceae SP264
Oceanospirillaceae NP32
Salinicola EAC73
Halomonas SAT64
Halomonas NAT30
Hahellaceae EAC91
Marinobacter IN6
Alteromonadaceae SP37
Alteromonadaceae CPC69
Gammaproteobacteria MED848
Gammaproteobacteria MED814
Gammaproteobacteria MED626
Sutterellaceae SAT62
Sutterellaceae NP16
Sutterellaceae ARS112
Cupriavidus NP124
SAR116 cluster MED717
SAR116 cluster MED595
SAR116 cluster MED139
Geminicoccus MED756
Sneathiella SAT66
Tistrella SP16
Tistrella EAC87
Tistrella CPC21
Thalassospira SP131
Thalassospira SAT2866
Thalassospira SAT187
Thalassospira SAT1308
Thalassospira SAT28
Thalassospira NP1073
Thalassospira IN19
Thalassospira EAC96
Thalassospira CPC43
Rhodospirillaceae SP4027
Rhodospirillaceae SP147
Rhodospirillaceae SAT2755
Rhodospirillaceae RS380
Rhodospirillaceae NP1282
Rhodospirillaceae NAT39
Rhodospirillaceae MED674
Rhodospirillaceae MED1079
Rhodospirillaceae EAC123
Nisaea MED620
Nisaea IN45
Magnetovibrio SP125
Magnetovibrio MED716
Rhodospirillaceae SP275
Rhodospirillaceae SP123
Rhodospirillaceae SAT9
Rhodospirillaceae SAT89
Rhodospirillaceae SAT126
Rhodospirillaceae NAT144
Rhodospirillaceae MED834
Rhodospirillaceae MED821
Rhodospirillaceae MED664
Rhodospirillaceae MED1257
Rhodospirillaceae IN18
Rhodospirillaceae EAC36
Rhodospirillales EAC21
Yangia NAT167
Stappia SP245
Stappia CPC38
Roseovarius SP300
Roseovarius SAT4
Roseovarius NAT17
Roseovarius EAC56
Roseobacter SP57
Roseobacter SP159
Roseobacter SAT8
Roseobacter NP7
Roseobacter NP10
Roseobacter MED633
Roseobacter EAC695
Roseobacter CPC4
Rhodovulum SAT37
Pelagibaca ARS5
Paracoccus SP169
Rhodobacteraceae MED215
Rhodobacteraceae ARS1029
Maritimibacter NAT7
Maritimibacter EAC76
Mameliella NP29
Confluentimicrobium SAT198
Ahrensia SAT55
Ahrensia NAT3
Rhodobacteraceae SP126
Rhodobacteraceae SAT57
Rhodobacteraceae NP49
Rhodobacteraceae NAT33
Rhodobacteraceae MED792
Rhodobacteraceae MED688
Rhodobacteraceae CPC40
Rhodobacteraceae CPC19
Rhodobiaceae MED879
Rhodobiaceae MED878
Rhodobacteraceae SP118
Nitratireductor NAT291
Phyllobacteriaceae SP17
Phyllobacteriaceae NAT20
Methylobacterium NP6
Filomicrobium MED665
Afipia MED728
Fulvimarina NAT173
Aurantimonas SAT5
Aurantimonas EAC15
Rhizobiales NAT353
Phenylobacterium IN8
Pelagibacterales sp. HTCC7211
Marinimicrobia MED866
Anaerolineaceae NAT117
Acidobacteria EAC88
MS NAO RS SAO IO SPO NPO SO
MS NAO RS SAO IO SPO NPO SO
NAO SAO IO SPO NPO SO

Figure S2. Taxon-specific enrichment of C-P lyase across depth zones and ocean regions. The heat map data presented was derived from mapping the raw reads of *Tara* Oceans metagenomes of the 0.22 micron size-fraction (Sunagawa *et al*., 2015) to metagenome-assembled genome (MAG) assemblies (Tully *et al*., 2018) containing C-P lyase. The analysis also included the genome assembly of *Pelagibacterales* sp. strain HTCC7211. The three panels (from left to right) correspond to metagenomes obtained from surface waters (5 m depth), the deep chlorophyll maximum (DCM), and the mesopelagic zone. The color scale bar indicates the average percentage of organisms possessing C-P lyase in each ocean region: Mediterranean Sea (MS), North Atlantic Ocean (NAO), Red Sea (RS), South Atlantic Ocean (SAO), Indian Ocean (IO), South Pacific Ocean (SPO), North Pacific Ocean (NPO), and Southern Ocean (SO). Note that the scale is in $\log_{10}$ units. Grey tiles indicate the mean percentage was equal to zero or could not be calculated due to the absence of recruited reads. The mean percentage of organisms represented by each MAG possessing C-P lyase (where 100% equals one copy per cell) was calculated by normalizing the coverage of *phnJ* sequences by the mean coverage of 40 single-copy marker genes (Sunagawa *et al*., 2013) and was averaged over the metagenomes belonging to the same ocean region. The upper panel depicts histograms of the distribution of the mean percentage of organisms of each MAG that possess C-P lyase across all ocean regions grouped by the corresponding depth zones. The left panel describes the phylogenetic classification of MAGs, mostly within the *Proteobacteria*. The colored tiles with Roman numerals on the left of the name of each MAG correspond to the color scheme of collapsed nodes in the PhnJ phylogenetic tree in Fig. S1. Blank spaces indicate that the corresponding MAG PhnJ sequence did not fall into a collapsed node in the tree.

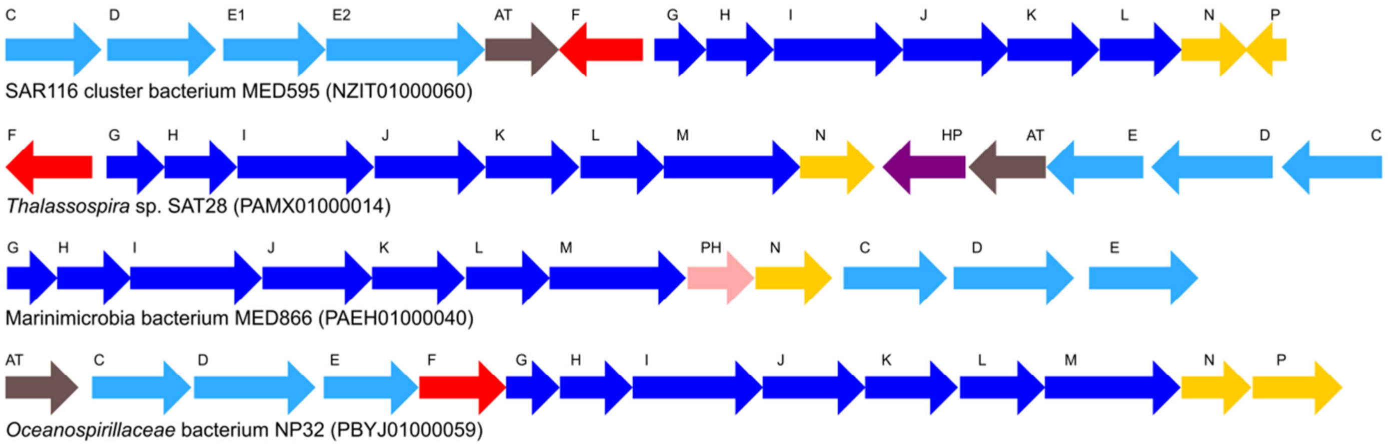

Figure S3. C-P lyase pathway *phn* gene operons from representative *Tara* Oceans metagenome assembled-genomes (MAGs). The bacterial lineages represented are the *Alphaproteobacteria* SAR116 cluster and *Rhodospirillaceae* (*Thalassospira* sp. SAT28), the candidate phylum Marinimicrobia, and the *Gammaproteobacteria* clade of *Oceanospirillaceae*. The functions encoded by each gene (arrows) are indicated in different colors: membrane transport, light blue; regulation, red; catalysis of C-P bond cleavage, blue; downstream processing, yellow; accessory, brown and violet. The arrow length is proportional to gene sequence length. The GenBank accession of each MAG contig containing the C-P lyase pathway is indicated in parentheses. Abbreviations: AT, putative acetyl transferase; HP, hypothetical protein; PH, putative phosphohydrolase.

References

Sunagawa, S., Coelho, L.P., Chaffron, S., Kultima, J.R., Labadie, K., Salazar, G., et al. (2015) Structure and function of the global ocean microbiome. *Science* **348**: 1261359.

Sunagawa, S., Mende, D.R., Zeller, G., Izquierdo-Carrasco, F., Berger, S.A., Kultima, J.R., et al. (2013) Metagenomic species profiling using universal phylogenetic marker genes. *Nat. Methods* **10**: 1196–1199.

Tully, B.J., Graham, E.D., and Heidelberg, J.F. (2018) The reconstruction of 2,631 draft metagenome-assembled genomes from the global oceans. *Sci. Data* **5**: 170203.
